# A. V. M. A.

**Published:** 1901-06

**Authors:** 


### A. V. M. A.

Every veterinarian should plan his visit to the Pan-American Exposition at Buffalo for the latter part of August or the first week in September, so as to attend the American Veterinary Medical Association's Convention at Atlantic City, N. J.

Dr. R. S. Huidekoper, of Philadelphia, Pa., will prepare a paper for the Atlantic City meeting. The subject will be announced in the next issue.

Dr. Lowe, of the Committee of Arrangements, has been lying seriously ill with articular rheumatism for the past four weeks. He is reported now as being convalescent.

Dr. Carl W. Gay, of Ithaca, N. Y., will present a paper at the Atlantic City meeting, and in all probability Dr. W. C. Fair, of Cleveland, Ohio, and Dr. George R. White, of Nashville, Tenn., will be represented on the programme.

